# TXNIP in liver sinusoidal endothelial cells ameliorates alcohol-associated liver disease via nitric oxide production

**DOI:** 10.7150/ijbs.90781

**Published:** 2024-01-01

**Authors:** Eunhye Jung, Eun Bok Baek, Eun-Ju Hong, Jee Hyun Kang, Suyoung Park, Sehee Park, Eui-Ju Hong, Young-Eun Cho, Je-Won Ko, Young-Suk Won, Hyo-Jung Kwon

**Affiliations:** 1 College of Veterinary Medicine, Chungnam National University, Daejeon 34134, Republic of Korea.; 2 Laboratory Animal Resource Center, Korea Research Institute of Bioscience and Biotechnology, Chungbuk 28116, Republic of Korea.; 3 Andong National University, Andong 36729, Republic of Korea.

**Keywords:** alcohol-associated liver disease, eNOS, liver sinusoidal endothelial cells, NO, TAK1, TXNIP

## Abstract

Dysregulation of liver sinusoidal endothelial cell (LSEC) differentiation and function has been reported in alcohol-associated liver disease (ALD). Impaired nitric oxide (NO) production stimulates LSEC capillarization and dysfunction; however, the mechanism underlying NO production remains unclear. Here, we investigated the role of thioredoxin-interacting protein (TXNIP), an important regulator of redox homeostasis, in endothelial cell NO production and its subsequent effects on ALD progression. We found that hepatic TXNIP expression was upregulated in patients with ALD and in ethanol diet-fed mice with high expression in LSECs. Endothelial cell-specific *Txnip* deficiency (*Txnip^ΔEC^*) in mice exacerbated alcohol-induced liver injury, inflammation, fibrosis, and hepatocellular carcinoma development. Deletion of *Txnip* in LSECs led to sinusoidal capillarization, downregulation of NO production, and increased release of proinflammatory cytokines and adhesion molecules, whereas *TXNIP* overexpression had the opposite effects. Mechanistically, TXNIP interacted with transforming growth factor β-activated kinase 1 (TAK1) and subsequently suppressed the TAK1 pathway. Inhibition of TAK1 activation restored NO production and decreased the levels of proinflammatory cytokines, thereby, blocking liver injury and inflammation in* Txnip^ΔEC^* mice*.* Our findings indicate that upregulated TXNIP expression in LSECs serves a protective role in ameliorating ALD. Enhancing TXNIP expression could, therefore, be a potential therapeutic approach for ALD.

## Introduction

Alcohol-associated liver disease (ALD) is a major cause of chronic liver disease that affects millions of patients worldwide. The pathological process of disease development involves early steatosis and steatohepatitis, with some individuals ultimately progressing to fibrosis/cirrhosis and even hepatocellular carcinoma (HCC) 1, 2. While disease progression and pathogenesis are relatively well understood, the exact mechanisms underlying ALD pathogenesis remain unclear, and there is currently no approved therapy to prevent or cure this disease.

Liver sinusoidal endothelial cells (LSECs) are specialized endothelial cells (ECs) that line the sinusoids. These highly endocytic ECs exhibit a characteristic phenotype of nondiaphramed fenestrae and lack a basal membrane, which facilitates highly efficient material exchange between the blood and space of Disse 3-5. Normally, LSECs increase nitric oxide (NO) production under shear stress in hepatic sinusoids to regulate blood flow and maintain their differentiated phenotype in an autocrine manner. Healthy differentiated LSECs maintain Kupffer cells (KCs) and hepatic stellate cells (HSCs) in a quiescent state through endothelial NO synthase (eNOS)-dependent NO pathways. On exposure to various damaging cues, such as oxidative stress and decreased NO bioavailability, LSECs dedifferentiate into a state called capillarization. Capillarized LSECs lose their fenestrae and express the extracellular matrix to develop basement membranes. This results in LSEC dysfunction, which contributes to the activation of KCs and HSCs and damage to hepatocytes. Altered LSECs release proinflammatory mediators and overexpress adhesion molecules, which then promote liver inflammation and fibrosis 3-5. Although LSEC capillarization and dysfunction are recognized as initial pathological markers in various liver diseases, including ALD 6, 7, the mechanisms underlying the impact of LSECs on the development of ALD remain unclear.

Thioredoxin-interacting protein (TXNIP), also known as thioredoxin-binding protein-2 (TBP-2) or vitamin D upregulated protein 1 (VDUP1), is a stress-response gene 8. TXNIP can induce oxidative damage by binding to thioredoxin (TRX) and inhibiting its antioxidant activity 9, 10. TXNIP is also linked to physiological processes related to cell growth, cell survival, and pathogenesis of various diseases 8. In the liver, TXNIP is significantly upregulated in metabolic dysfunction-associated steatotic liver disease (MASLD) patients and mediates lipogenesis and fatty acid re-esterification 11, 12. In a previous study, we demonstrated that TXNIP is upregulated in patients with MASLD and elevated TXNIP attenuated steatohepatitis via autophagy and fatty acid oxidation 13. In addition, TXNIP expression is increased in the liver exposed to ethanol, suggesting that TXNIP may be involved in the pathogenesis of ALD 14.

Recently, TXNIP was shown to mediate EC inflammation in response to disturbed blood flow by increasing monocyte adhesion 15. It also triggers early apoptosis in high-glucose-treated human aortic ECs 16, 17, and its upregulated expression contributes to oxidative stress and endothelial dysfunction in hypertensive rats 17, 18. Furthermore, TXNIP can suppress eNOS activity and disrupt NO signaling 19, 20, indicating that it negatively regulates endothelial function. However, little is known about the role of TXNIP in LSECs during the development of liver diseases. Here, we aimed to address this question by determining how exposure to alcohol affects TXNIP expression in LSECs in patients with ALD and by evaluating the function of TXNIP in ALD development with regard to NO production using mice genetically lacking *Txnip* in LSECs and a human LSEC line overexpressing *TXNIP*.

## Materials and methods

### Human liver samples

Tissue microarrays containing ALD specimens were purchased from SEKISUI XenoTech (Kansas City, KS, USA). Each microarray contained 13 healthy human liver tissue samples (TMA. NORM) from donors without a history of alcohol consumption and 19 and 18 samples with steatosis (TMA. AS) and fibrosis (TMA. FIB), respectively, both from donors with a history of alcohol use. The general characteristics of healthy and ALD human liver samples are listed in the Supporting Information (Table S1). The human study was approved by the Chungnam National University Institutional Review Board (Approval Number: 02205-BR-061-01) and was performed in accordance with the Declaration of Helsinki and principles set out in the Department of Health and Human Services Belmont Report.

### Animal experiments

*Txnip^-/-^* mice on a C57BL/6J background were described previously 13. C57BL/6J mice were used as controls. Macrophage- or EC-specific *Txnip*-deficient (*Txnip^ΔMac^* and* Txnip^ΔEC^*) mice were generated by crossing *Txnip^fl/fl^* mice with *LysM-Cre* and* Tie2-Cre* transgenic mice (The Jackson Laboratory, Bar Harbor, MN, USA), respectively. Animals were maintained on a standard rodent chow diet with a 12 h light/dark cycle. All animal studies were approved by the Chungnam National University Animal Care and Use Committee (Approval Numbers: 202103A-CNU-093 and 202203A-CNU-012) and conducted according to the principles and procedures outlined in the National Institutes of Health Guide for the Care and Use of Laboratory Animals.

### Mouse models for ethanol consumption

Eight-week-old female or male wild-type (WT),* Txnip^-/-^*, *Txnip^ΔMac^*,* Txnip^ΔEC^*, and* Txnip^fl/fl^* mice were fed either a Lieber-DeCarli liquid diet containing 4% or 5% ethanol (ethanol-fed) or liquid control diet (pair-fed) for 1 year or 4 weeks. Mice were initially fed a Lieber-DeCarli diet (Dyets, Bethlehem, PA, USA) *ad libitum* for 5 days to adapt them to a liquid diet, followed by a gradual increase in ethanol (Sigma-Aldrich, Burlington, MA, USA) content by 1% (v/v) each day until it reached 4% or 5% (v/v). The mice were then fed this liquid diet for 1 year (4%) or 4 weeks (5%). In some experiments, mice were fed 5% ethanol for 10 days, followed by one ethanol binge (5 g/kg, referred to as the chronic binge model), as described previously 21. For NG25 treatment, *Txnip^ΔEC^* and* Txnip^fl/fl^* mice were fed a liquid diet containing 5% ethanol for 4 weeks, with further administration of NG25 (5 mg/kg/day intraperitoneally; MedChemExpress, Monmouth Junction, NJ, USA) or vehicle.

### Histological examination

Formalin-fixed liver samples were paraffin embedded, sectioned, and stained with hematoxylin and eosin (H&E; Labcore, Seoul, Korea). In Sirius red (IHC World, Woodstock, MD, USA) staining, positive area was calculated from at least five magnification (final magnification, ×400) fields per liver section.

### Blood chemistry and hepatic lipid levels

Serum alanine aminotransferase (ALT) and aspartate aminotransferase (AST) levels were determined using a HITACHI 7020 chemistry analyzer (Hitachi, Tokyo, Japan). Hepatic cholesterol and triglyceride (TG) content were determined using a cholesterol quantitation kit (BioVision, Waltham, MA, USA) and a TG colorimetric assay kit (Cayman, Ann Arbor, MI, USA), respectively following manufacturer's instructions.

### Immunohistochemistry (IHC) analysis

Paraffin-embedded liver tissues from human and mice were used for IHC analyses. Briefly, tissues were incubated with a primary antibody against TXNIP, F4/80, myeloperoxidase (MPO), CD31 (Abcam, Cambridge, United Kingdom), or LYVE1 (R&D Systems, Minneapolis, MN, USA) followed by incubation with the corresponding biotinylated secondary antibody (Vector Laboratories, Newark, CA, USA) (Table S2). For analysis of TXNIP expression, five fields (final magnification, ×200) were randomly selected for each slide, and the integrated optical density (IOD) of all positive staining in each image was measured using the MetaMorph 4.6 imaging software system (Molecular Devices, San Jose, CA, USA). F4/80- or MPO-positive cells were counted in 10 randomly selected fields per section (final magnification, ×400).

### Immunofluorescence (IF) analysis

Human and mouse liver sections were incubated with a primary antibody against TXNIP, F4/80, CD31 (Abcam), TAK1 (Cell Signaling Technology, Danvers, MA, USA), or LYVE1 (R&D Systems) followed by incubation with a fluorescence-conjugated antibody (Invitrogen, Waltham, MA, USA) (Table S2). The LYVE-1- or CD31-positive area was analyzed in 10 randomly selected fields per section (final magnification, ×400).

### Malondialdehyde (MDA) assay

Hepatic MDA levels were determined using a TBARS assay kit (Cell Biolabs, San Diego, CA, USA), according to the manufacturers' instructions.

### Isolation and treatment of primary mouse cells

Primary mouse hepatocytes, HSCs, KCs, and LSECs were isolated from *Txnip^-/-^* and WT mice as previously described 22. Briefly, after collagenase perfusion of liver, the cell suspension was centrifuged at 50 × *g* for 5 min to obtain hepatocytes (pellet fraction). The non-parenchymal cell (NPC)-containing supernatant was separated on 11.5% or 20% Opti-Prep gradients (Sigma-Aldrich) for isolation of HSC and KC/LSEC fractions, respectively. Each LSEC-enriched fraction was incubated with anti-CD146 magnetic beads (Miltenyi Biotec, Bergisch Gladbach, Germany) and the mixture was applied to the magnetic column of a magnetic-activated cell sorting (MACS) separator. Positively selected LSECs were cultured in EGMTM-2 MV Microvascular Endothelial Cell Growth Medium-2 supplemented with an EGMTM-2 MV SingleQuots^TM^ Supplement Pack (EBM-2; Lonza, Basel, Switzerland) at 37°C in a humidified 5% CO_2_ atmosphere. Negatively selected KCs were cultured in DMEM supplemented with 10% fetal bovine serum (FBS) and 1% penicillin and streptomycin at 37°C.

### Human LSEC line

The immortalized human LSEC line, TMNK-1, was purchased from SEKISUI XenoTech. Cells were cultured in MCDB 131 (Gibco, Waltham, MA, USA) supplemented with an EGMTM-2 Endothelial SingleQuots^TM^ Kit (Lonza). To establish stable cell lines overexpressing *TXNIP*, TMNK-1 cells were transfected with control or *TXNIP*-expressing constructs (EX-M0226-Lv105 and EX-NEG-M105, GeneCopoeia, Rockville, MD, USA) using the Xfect transfection reagent (Clontech Laboratories, Mountain View, CA, USA) according to the manufacturer's recommendations.

### Lipopolysaccharide (LPS) treatment of cells

For LPS treatment, mouse primary LSECs or TMNK-1 cells were treated with LPS (0.75 μg/ml; Sigma-Aldrich) for the indicated time periods. For TAK1 inhibitor treatment, NG25 (500 nM; Selleck Chemicals, Houston, TX, USA) was pretreated for 3 h, followed by treatment with LPS (0.75 μg/ml) for an additional 6 or 24 h.

### Western blot analysis

Equal amounts of protein were separated using SDS-PAGE and transferred onto a nitrocellulose membrane. The membranes were incubated with antibodies against phospho-eNOS, total-eNOS, phospho-TAK1, total-TAK1, phospho-JNK, total-JNK, phospho-p38 MAPK, total-p38 MAPK, phospho-ERK1/2, total-ERK1/2 (Cell Signaling Technology), and β-actin (Sigma-Aldrich) (Table S2). The relative density was calculated as the ratio of the intensity of the protein of interest to that of β-actin, and all band intensities were within the linear range.

### RNA isolation and real-time PCR (qRT-PCR)

Total RNA was extracted from mouse liver tissue and cells using TRIzol (Thermo Fisher Scientific, Waltham, MA, USA) according to the manufacturer's instruction. Expression levels of target genes were quantified by performing qRT-PCR analysis with *gapdh* used as an internal control. The fold change in mRNA level was expressed as 2^-ΔΔCt^. Primer pairs used for qRT-PCR are listed in Supporting Information (Table S3).

### NO assay

Intracellular NO levels in cells were measured using a Nitric Oxide Assay Kit (Abcam) according to the manufacturer's instructions. Briefly, cells were resuspended in warm culture medium and NO Red Dye stock solution was added into the culture medium. NO formation was induced by treating stained cells with LPS and incubating them at 37°C in a 5% CO_2_ incubator for 6 or 24 h. Increase in fluorescence was monitored using a flow cytometer (BD FACSCanto™ System; BD Bioscience, San Jose, CA, USA) at excitation and emission wavelengths of 630 and 660 nm, respectively, and analyzed using the BD FACSDiva™ software (BD Bioscience).

### Immunoprecipitation (IP)

For IP, the TAK1 plasmid was transfected into *TXNIP*-overexpressing TMNK-1 cells and positive clones were screened on G-418 (Thermo Fisher Scientific) for 3 weeks. The TAK1 cloning plasmid, pcDNA3 TAK1/F, was provided by Xin Lin (Addgene plasmid). IP was performed using the Dynabead Protein A immunoprecipitation kit (Thermo Fisher Scientific).

### RNA-Seq and analysis

RNA was obtained from LSECs isolated from *Txnip^-/-^* and WT mice. Total RNA was isolated using the TRIzol reagent (Thermo Fisher Scientific). For control and test RNAs, library was constructed using a QuantSeq 3′ mRNA-Seq Library Prep Kit (Lexogen, Vienna, Austria) according to the manufacturer's instructions. High-throughput sequencing was performed as single-end 75 sequencing using a NextSeq 550 (Illumina, Inc., San Diego, CA, USA). QuantSeq 3′ mRNA-Seq reads were aligned using Bowtie2 23. Differentially expressed genes (DEGs) were determined based on counts from unique and multiple alignments using coverage in Bedtools 24. Gene classification was based on searches done against DAVID (https://david.ncifcrf.gov/) and Medline databases (http://www.ncbi.nlm.nih.gov/). Data mining and graphic visualization were performed using ExDEGA (Ebiogen). DEGs were identified using the following criteria: log fold change > 1.3 and corrected *p* value < 0.05. The RNAseq data described herein have been deposited in NCBI Gene Expression Omnibus and are accessible through the GEO Series accession number GSE217238 (https://www.ncbi.nlm.nih.gov/geo/query/acc.cgi?acc=GSE217238).

### Statistical analysis

Data are presented as means ± standard deviation (SD). Student's *t*-test was used to compare the values between the two groups. Values obtained from three or more groups were compared using one-way analysis of variance, followed by Tukey's *post-hoc* test. The relationship between TXNIP expression and clinicopathological features was analyzed using the chi-square test. The incidence of hepatic tumors was evaluated using the Fisher's exact test. Statistical analyses were performed using the GraphPad Prism software (GraphPad Software, San Diego, MA, USA). Statistical significance was set at *p* < 0.05.

## Results

### TXNIP expression is upregulated in the liver of patients with ALD and ethanol diet-fed mice

**Figure 1:**
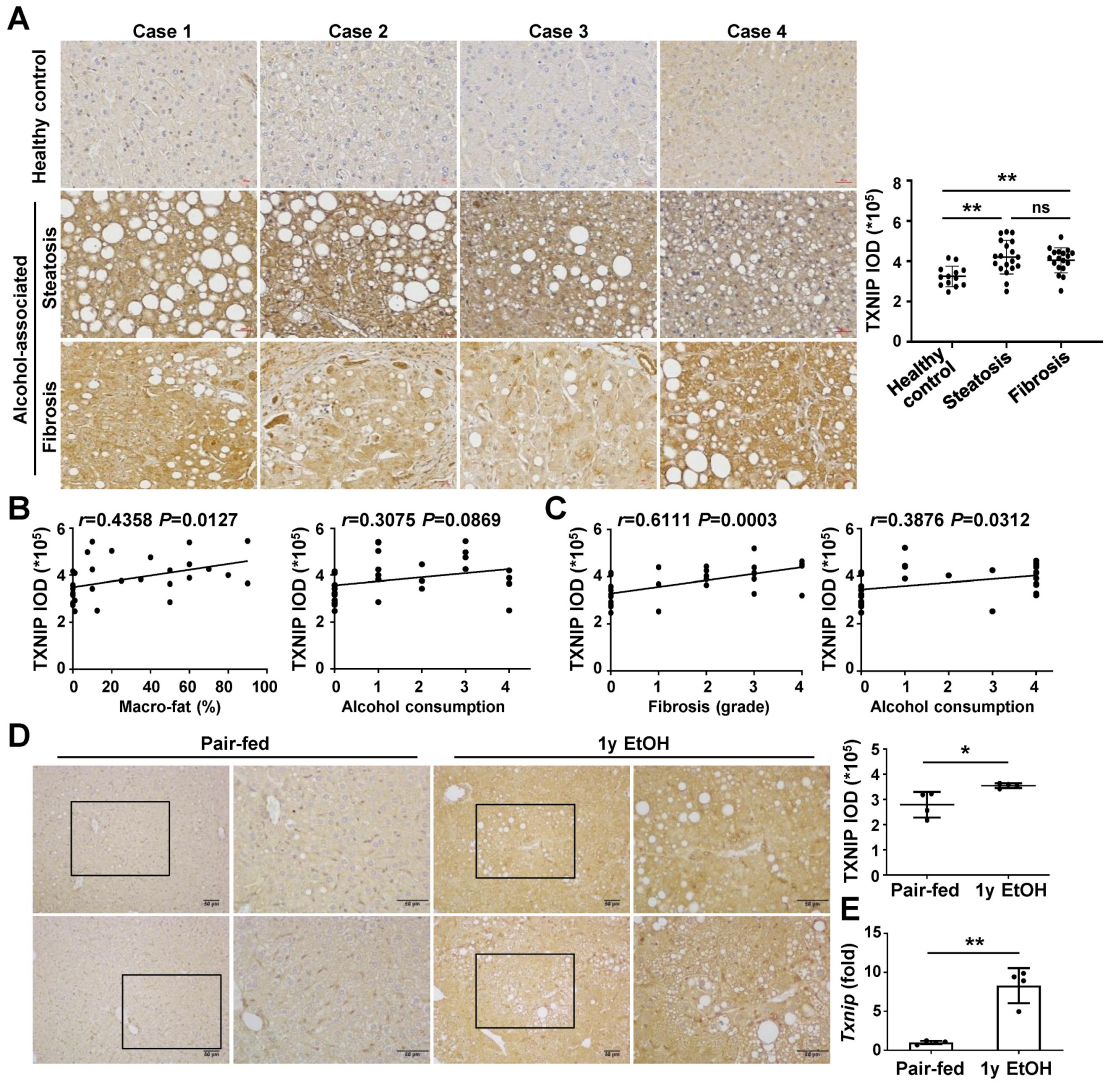
** TXNIP expression is increased in the liver tissue of patients with ALD and ethanol diet-fed mice. (A)** Representative image of immunohistochemical staining of TXNIP in liver sections from healthy controls (n = 13), patients with alcohol-associated steatosis (n = 19), and patients with alcohol-associated fibrosis (n = 18). Original magnification, ×200. The integrated optical density (IOD) of TXNIP-positive areas is shown on the right. **(B)** Association between TXNIP expression and macro-fat or alcohol consumption in ALD patients with steatosis. Alcohol consumption was defined as follows: Heavy (grade 4) > Moderate (grade 3) > Social (grade 2) > Occasional (grade 1) > None (grade 0).** (C)** Correlation between TXNIP expression and fibrosis grade or alcohol consumption in ALD patients with fibrosis. **(D)** Representative immunohistochemical images of TXNIP expression in mice fed a control or 4% ethanol diet for 1 year (1y EtOH, n = 4). Scale bars, 50 μM. The IOD of TXNIP-positive areas is shown on the right. **(E)** Hepatic expression of *Txnip* mRNA in mice fed a control or 4% ethanol diet for 1 year (1y EtOH, n = 4). Data are presented as means ± SD. *p < 0.05; **p < 0.01. “ns” stands for “not significant.”

To analyze whether TXNIP expression is associated with the development of ALD, we first examined the hepatic levels of TXNIP in patients with ALD. IHC revealed markedly increased TXNIP expression in patients with alcohol-associated steatosis and fibrosis compared with that in healthy controls (Fig. 1A). The degree of TXNIP expression was positively correlated with the percentage of macro-fat in alcoholic patients with steatosis (Fig. 1B). Correlation analysis also showed a significant association between increased TXNIP expression and the severity of fibrosis or ethanol intake in alcoholic patients with fibrosis (Fig. 1C). Consistent with the findings in human samples, the protein and mRNA expression levels of TXNIP were significantly increased in mice fed the ethanol diet (Fig. 1D and E). Taken together, these findings suggest that TXNIP overexpression is related to ALD and that TXNIP plays an important role in this process.

### Endothelial-specific deletion of Txnip exacerbates ethanol-induced liver injury, inflammation, fibrosis, and HCC development

To investigate how TXNIP contributes to the development of ALD, *Txnip* total knockout (*Txnip^-/-^*) and WT mice were fed a control or 4% ethanol diet for 1 year. Ethanol-fed *Txnip^-/-^* mice had a lower survival rate, higher liver-to-body weight ratio, and higher serum levels of ALT and AST than did ethanol-fed WT mice (Fig. S1A-C). We found that 3 of 9 ethanol-fed *Txnip^-/-^* mice (33.3%) developed tumor nodules of variable sizes in the liver, whereas no obvious hepatic tumor was observed in ethanol-fed WT mice (Fig. S1D-F). In addition, ethanol-fed *Txnip^-/-^* mice exhibited an increased number of F4/80- and MPO-positive cells and Sirius red-positive areas (Fig. S1G). qRT-PCR analysis showed that the hepatic expression of genes involved in inflammation (tumor necrosis factor-α [*Tnf*], interleukin-6 [*Il6*], IL-1β [*Il1b*], and C-C motif chemokine ligand 2 [*Ccl2*]) and fibrosis (transforming growth factor-β [*Tgfb1*], collagen 1 α1 [*Col1a1*], and tissue inhibitor of metalloproteinases-1 [*Timp1*]) were markedly elevated in *Txnip^-/-^* mice (Fig. S1H and I).

**Figure 2:**
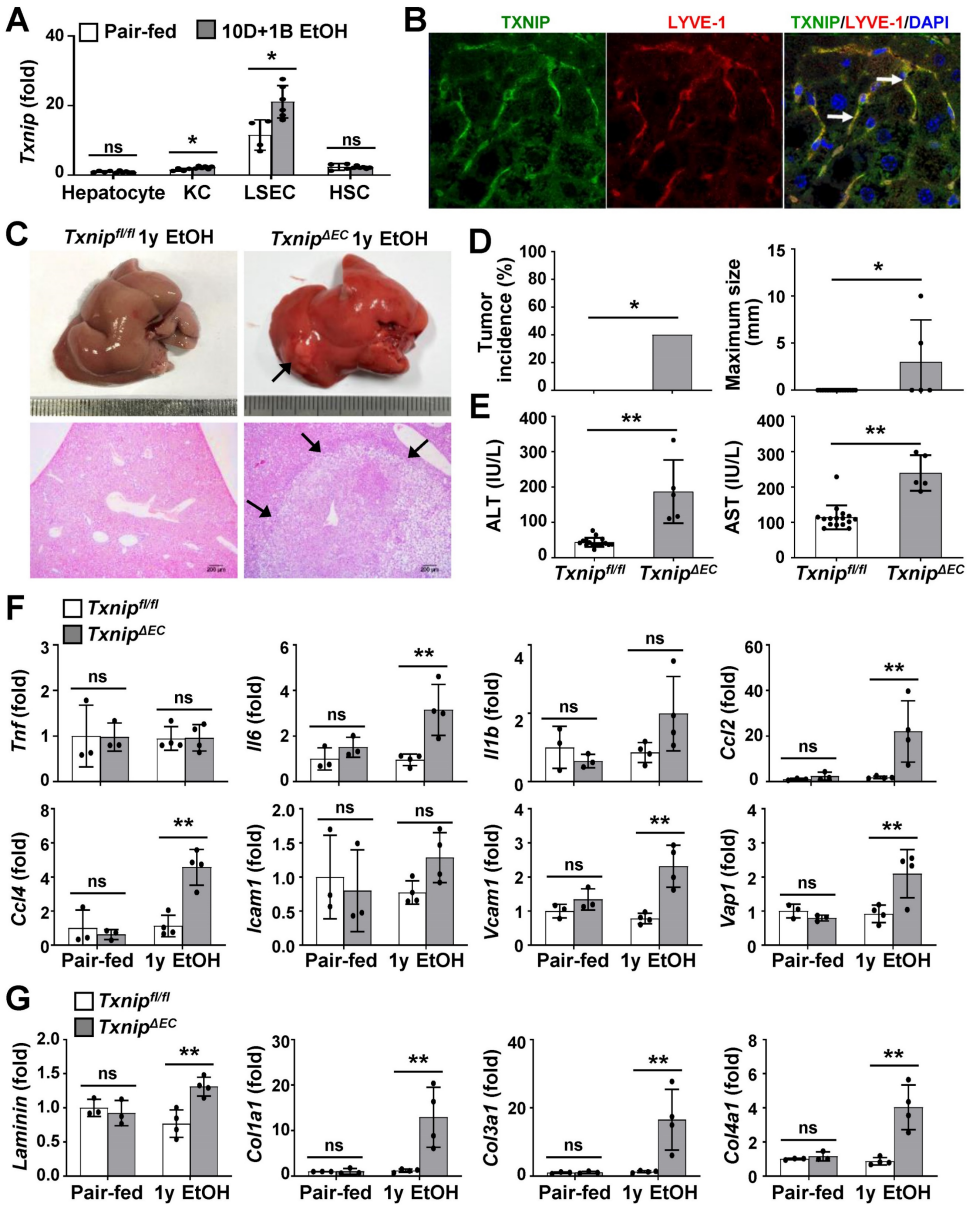
** Deficiency of *Txnip* in LSECs increases ethanol-induced liver diseases. (A)** Relative mRNA levels of *Txnip*. Hepatocytes, KCs, LSECs, and HSCs were isolated from WT mice after 10 day plus gavage (10D+1B EtOH; pair-fed, n = 4; ethanol [EtOH], n = 6). **(B)** Immunofluorescence staining of WT mice. Note the colocalization of TXNIP and LYVE-1 (arrows). Original magnification, ×400. **(C-G)** *Txnip^fl/fl^* and *Txnip^ΔEC^* mice were fed a liquid control diet or 4% ethanol diet for 1 year (1y EtOH). Representative gross findings and H&E staining of liver sections (C), incidence and maximum size of tumors (D), serum levels of ALT and AST (E) (*Txnip^fl/fl^*, n = 16; *Txnip^ΔEC^*, n =5), and qRT-PCR analysis (F-G) (pair-fed, n = 3; EtOH, n = 4). Note tumor nodule (arrows) compressing adjacent normal liver tissue in *Txnip^ΔEC^* mice. Scale bars, 200 μM. Data are presented as means ± SD. *p < 0.05; **p < 0.01. “ns” stands for “not significant.”

Because TXNIP was upregulated in patients with ALD and in ethanol-fed mice, we next investigated the cell types responsible for this effect. Hepatocytes, KCs, LSECs, and HSCs were isolated from chronic binge ethanol-fed WT mice, and TXNIP expression was analyzed. In the control diet-fed WT mice, the major *Txnip*-expressing cells were LSECs, which exhibited approximately 11.5-, 7.49-, and 4.89-fold higher *Txnip* expression than did hepatocytes, KCs, and HSCs, respectively (Fig. 2A). Chronic binge ethanol feeding increased *Txnip* mRNA levels in KCs (1.4-fold) and LSECs (1.8-fold), but not in hepatocytes and HSCs (Fig. 2A). We also co-stained liver sections for TXNIP and markers of KCs (F4/80) or LSEC (lymphatic vessel endothelial hyaluronan receptor 1; LYVE-1) and found that TXNIP was highly expressed in KCs and LSECs (Fig. 2B and Fig. S2A).

To determine which hepatic cell type contributed to alcohol-induced liver diseases by expressing TXNIP, we generated macrophage- or EC-specific *Txnip*-deficient (*Txnip^ΔMac^* and* Txnip^ΔEC^*) mice by crossing *Txnip^fl/fl^* mice with *LysM-Cre* and* Tie2-Cre* transgenic mice, respectively. Loss of *Txnip* expression was confirmed by nondetectable* Txnip* mRNA levels in KCs and LSECs isolated from *Txnip^ΔMac^* or* Txnip^ΔEC^* mice compared with those in control (*Txnip^fl/fl^*) mice (Fig. S2B). We then determined the effects of KC or LSEC *Txnip* depletion on ALD development. Compared to *Txnip^fl/fl^* mice, *Txnip^ΔEC^* mice had higher levels of serum ALT and AST and hepatic expression of *Tnf*,* Il6*,* Il1b*, and adhesion G protein-coupled receptor E1 (*Adgre1*) after 4 weeks of alcohol feeding; however, there was no significant difference in hepatic TG or cholesterol levels (Fig. S2C-E). *Txnip^ΔMac^* mice showed no change in liver injury or inflammatory response compared with *Txnip^fl/fl^* mice (Fig. S2C-E). We further examined whether the increased liver injury and inflammation in *Txnip^ΔEC^* mice were associated with HCC development. After 1 year of ethanol feeding, hepatic tumorigenesis was strongly accelerated in *Txnip^ΔEC^* mice compared with that in *Txnip^fl/fl^* mice (Fig. 2C and D) and was associated with increased liver damage (serum ALT and AST levels and liver histology), inflammation (the number of MPO-positive cells and expression levels of *Il6*, *Ccl2*, *Ccl4,* vascular cell adhesion molecule 1 [*Vcam1*], and vascular adhesion protein 1 [*Vap1*]), and fibrosis (the Sirius red-stained area and expression levels of *Laminin*,* Cola1*,* Col3a1*, and *Col4a1*) (Fig. 2E-G and Fig. S3). These results indicate that endothelium-specific deletion of *Txnip* likely increases susceptibility to ethanol-induced liver injury, inflammation, fibrosis, and HCC development.

### Endothelial Txnip deficiency reduces NO production and promotes LSEC capillarization and proinflammatory function

**Figure 3:**
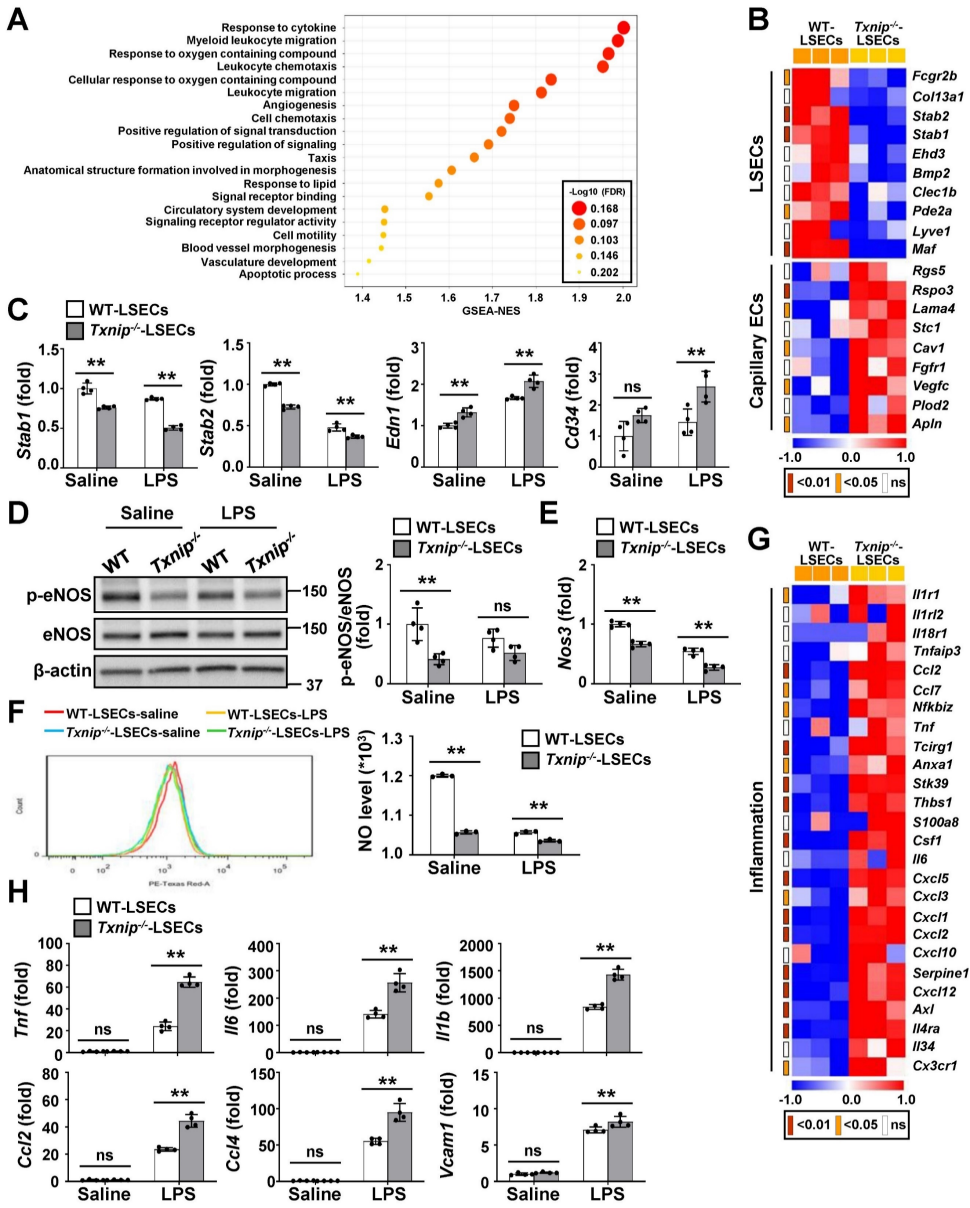
** TXNIP regulates LSEC capillarization, NO production, and proinflammatory function. (A)** GSEA of RNA-seq data. RNA samples were collected from LSECs isolated from *Txnip^-/-^* and WT mice (n = 3). The 20 most significantly enriched pathways are shown. **(B)** Heat maps showing changes in the expression of mRNAs related to LSEC differentiation (n = 3). The *p* values for the comparisons are indicated in color. **(C)** Relative mRNA levels of LSECs and capillary EC markers (n = 4). LSECs isolated from *Txnip^-/-^* and WT mice treated with LPS (0.75 μg/ml) for 6 h. **(D-F)** LSECs isolated from *Txnip^-/-^* and WT mice were incubated with LPS (0.75 μg/ml) for 6 h and subjected to western blotting (D) (n = 4), qRT-PCR analysis (E) (n = 4), and NO assay (F) (n = 3). **(G)** Heat maps showing changes in the expression of mRNAs related to inflammation (n = 3). The *p* values for the comparisons are indicated in color. **(H)** Relative mRNA levels of genes related to inflammation. LSECs isolated from *Txnip^-/-^* and WT mice treated with LPS (0.75 μg/ml) for 6 h. Data are presented as means ± SD. *p < 0.05; **p < 0.01. “ns” stands for “not significant.”

To evaluate the effects of TXNIP on LSECs, we performed RNA-seq on LSECs isolated from *Txnip^-/-^* and WT mice (Fig. S4A). The two groups were clearly separated in the cluster analysis, indicating that they differed significantly (Fig. S4B). Gene set enrichment analysis (GSEA) revealed that cellular signaling pathways or genes related to inflammation, angiogenesis, and vasculature morphogenesis/development were significantly affected by *Txnip* deficiency (Fig. 3A). A heat map revealed that *Txnip*-deficient LSECs exhibited downregulation of LSEC-associated genes and upregulation of capillary EC-associated genes (Fig. 3B). qRT-PCR confirmed that the capillary EC markers, endothelin-1 (*Edn1*) and *Cd34*, were upregulated in *Txnip*-deficient LSECs, whereas the LSEC differentiation markers, stabilin-1 (*Stab1*) and stabilin-2 (*Stab2*), were downregulated (Fig. 3C). LPS treatment promoted LSEC capillarization, which was much greater in *Txnip*-deficient LSECs (Fig. 3C). Consistently, 1 year of ethanol feeding significantly decreased LSEC-associated protein (LYVE-1) expression in *Txnip^ΔEC^* mice but increased the protein and mRNA levels of capillary EC-related factors (*Cd31*, *Cd34*, and *Edn1*) (Fig. S4C and D). The eNOS-NO signaling pathway maintains the LSEC-differentiated phenotype, and dysfunctional LSECs exhibit impaired NO production 25. The protein expression of phospho-eNOS, mRNA level of endothelial NO synthase (*Nos3*), and NO production were lower in *Txnip*-deficient LSECs than in WT LSECs under normal culture conditions and after LPS treatment (Fig. 3D-F). Furthermore, 1 year ethanol-fed *Txnip^ΔEC^* mice showed significantly decreased expression of eNOS versus *Txnip^fl/fl^* mice (Fig. S4E).

Capillarized and dysfunctional LSECs acquire a proinflammatory phenotype and function via the release of proinflammatory mediators and overexpression of adhesion molecules 3. Analysis of RNA-seq data indicated that a set of genes involved in inflammation was altered upon *Txnip* knockout (Fig. 3G). Consistently, *Txnip*-deficient LSECs showed upregulation of *Tnf*,* Il6*,* Il1b*,* Ccl2*,* Ccl4*, and *Vcam1* compared to WT LSECs after LPS treatment (Fig. 3H). Additionally, LSECs isolated from chronic binge ethanol-fed *Txnip^ΔEC^* mice exhibited increased expression of *Tnf*,* Il6*,* Il1b*, and *Ccl2* (Fig. S4F). Collectively, these results suggest that the lack of endothelial *Txnip* results in impaired NO production and an altered LSEC phenotype, which might promote liver injury and inflammation.

### Endothelial TXNIP overexpression inhibits LSEC capillarization and stimulates NO production

**Figure 4:**
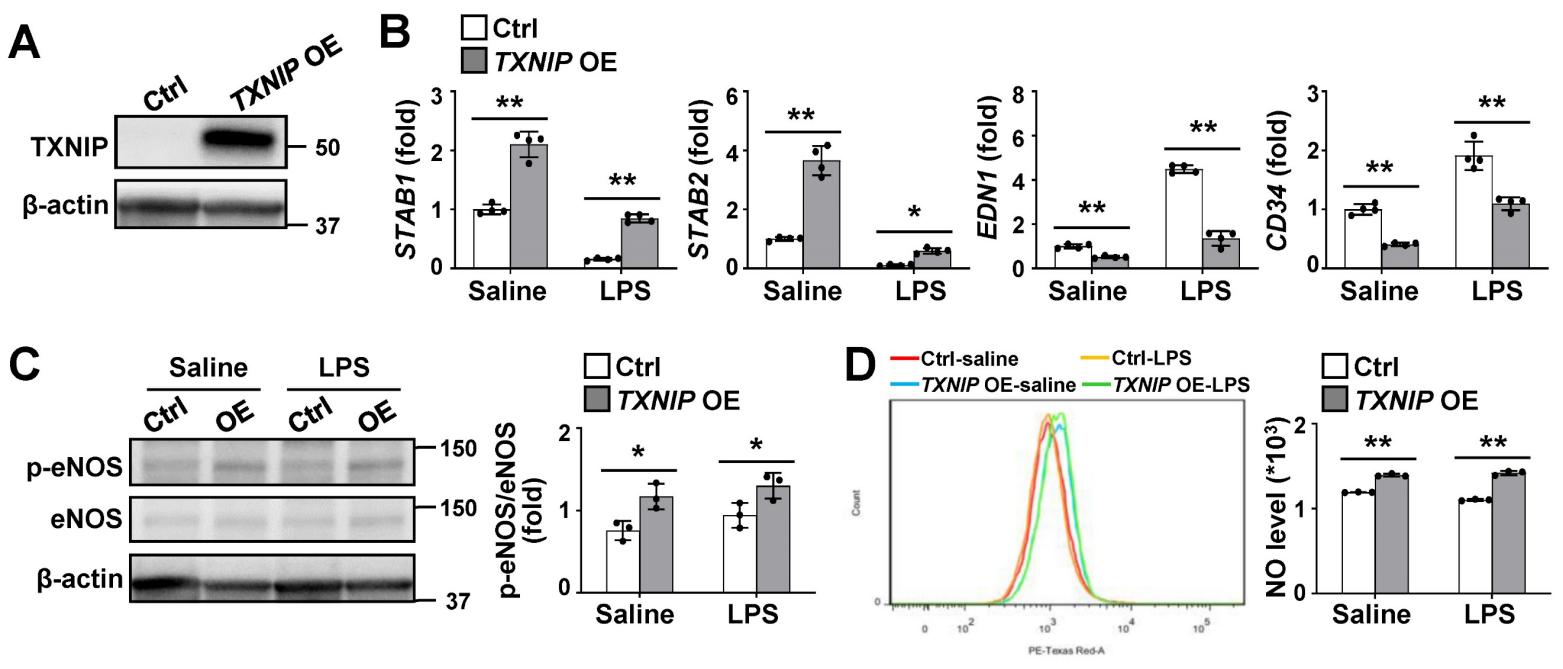
** Endothelial *TXNIP* overexpression blocks LSEC capillarization and increases NO production. (A)** Western blot analysis of TXNIP in control (Ctrl) and *TXNIP*-overexpressing (*TXNIP* OE) TMNK-1 cells. **(B)** Relative mRNA levels of LSECs and capillary EC markers (n = 4 biological replicates). Ctrl and *TXNIP*-OE TMNK-1 cells were incubated with saline or LPS (0.75 μg/ml) for 24 h. **(C-D)** Ctrl and *TXNIP*-OE TMNK-1 cells were incubated with saline or LPS (0.75 μg/ml) for 24 h and subjected to western blotting (C) and NO assay (D) (n = 3 biological replicates). Data are presented as means ± SD. *p < 0.05; **p < 0.01.

To directly determine the functional role of TXNIP in LSECs, we established stable *TXNIP*-overexpressing (*TXNIP*-OE) TMNK-1 cells, a human LSEC line and verified using western blotting (Fig. 4A). qRT-PCR analysis revealed that the expression levels of *STAB1* and *STAB2* were much higher in *TXNIP*-OE TMNK-1 cells than in vector control cells under normal culture conditions, whereas *EDN1* and *CD34* levels were downregulated (Fig. 4B). In addition, LPS-induced increase in *EDN1* and *CD34* was suppressed in *TXNIP*-OE TMNK-1 cells, whereas LPS-induced decrease in the levels of *STAB1* and *STAB2* was partially inhibited by *TXNIP* overexpression (Fig. 4B). Compared with vector control, *TXNIP* overexpression increased the phospho-eNOS level and NO production under normal culture conditions and after LPS treatment (Fig. 4C and D). Collectively, these findings demonstrate that endothelial *TXNIP* overexpression prevents LSEC capillarization and increases NO level.

### TXNIP interacts with TAK1 and inhibits TAK1/JNK signaling in LSECs

**Figure 5:**
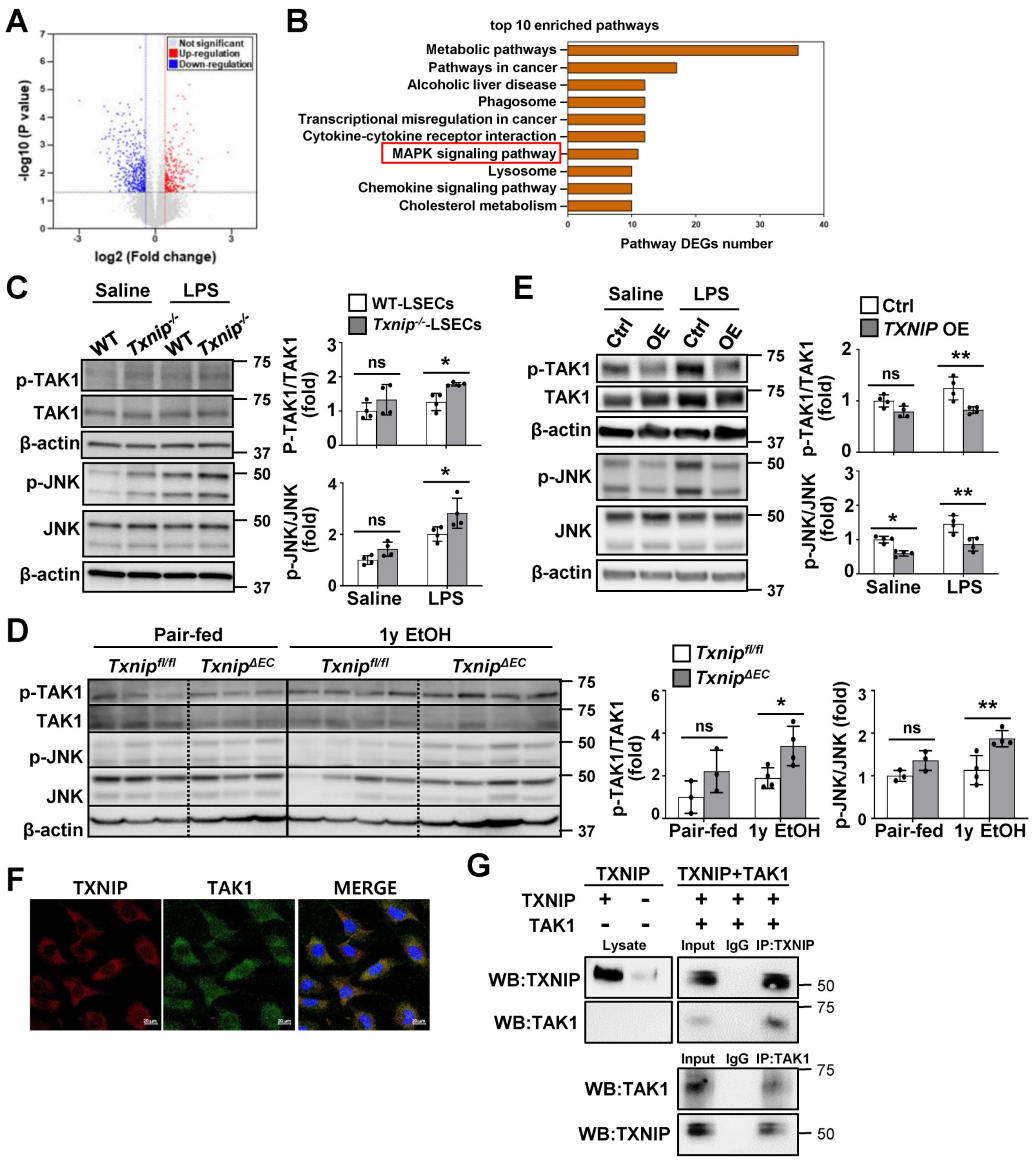
** TXNIP inhibits TAK1/JNK signaling in LSECs. (A)** Volcano map showing DEGs in LSECs isolated from *Txnip^-/-^* and WT mice (n = 3). **(B)** The top-10 most significantly enriched pathways contributing to TXNIP function based on the KEGG enrichment analysis. **(C)** Protein expression of TAK1 and JNK. LSECs isolated from *Txnip^-/-^* and WT mice were incubated with LPS (0.75 μg/ml) for 30 min (n = 4). **(D)** Western blot analysis of the liver tissue (pair-fed, n = 3; ethanol [EtOH], n = 4). *Txnip^fl/fl^* and* Txnip^ΔEC^* mice were fed a control or 4% ethanol diet for 1 year (1y EtOH). **(E)** Western blot analysis (n = 4 biological replicates). Control (Ctrl) and *TXNIP*-overexpressing (*TXNIP* OE) TMNK-1 cells were incubated with LPS (0.75 μg/ml) for 30 min. **(F)** Immunofluorescence staining of TXNIP and TAK1 in TMNK-1 cells. Original magnification, ×400. Scale bars, 20 μM. **(G)** Representative coimmunoprecipitation analysis of TXNIP and TAK1 in TMNK-1 cells. Data are presented as means ± SD. *p < 0.05; **p < 0.01. “ns” stands for “not significant.”

To investigate the mechanism by which TXNIP regulates LSECs, we searched for DEGs by analyzing RNA-seq data from the LSECs of *Txnip^-/-^* and WT mice. We identified 326 DEGs, of which 139 were upregulated and 187 were downregulated, in *Txnip*-deficient versus WT LSECs (Fig. 5A). The Kyoto encyclopedia of genes and genomes (KEGG) pathway enrichment analysis showed that the mitogen-activated protein kinase (MAPK) pathway was most significantly altered by *Txnip* deficiency (Fig. 5B). Western blot analysis further revealed that LPS treatment elevated TAK1 and JNK phosphorylation, which was greatly increased in *Txnip*-deficient LSECs (Fig. 5C). TAK1 and JNK activation was also increased in ethanol-fed *Txnip^ΔEC^* mice compared with that in *Txnip^fl/fl^* mice (Fig. 5D). In contrast, *TXNIP* overexpression inhibited LPS-induced phosphorylation of TAK1 and JNK (Fig. 5E). *Txnip* deficiency or overexpression in LSECs affected the phosphorylation of p38 MAPK and extracellular signal-regulated kinase (ERK) *in vitro*. However, p38 MAPK and ERK signals did not change in ethanol-fed *Txnip^ΔEC^* mice (Fig. S5). To further investigate the potential molecular mechanisms by which TXNIP regulates the MAPK pathway in LSECs, we examined the localization of TXNIP and TAK1. IF staining showed that TXNIP colocalized with TAK1 in the cytoplasm of TMNK-1 cells (Fig. 5F). Co-IP experiments revealed that TXNIP interacted with TAK1 in TMNK-1 cells (Fig. 5G). Taken together, these findings demonstrate that TXNIP interacts with TAK1 and regulates TAK1/JNK pathway in LSECs.

### Inhibition of TAK1 activity restores NO production and blocks ethanol-induced liver injury and inflammation in Txnip^ΔEC^ mice

**Figure 6:**
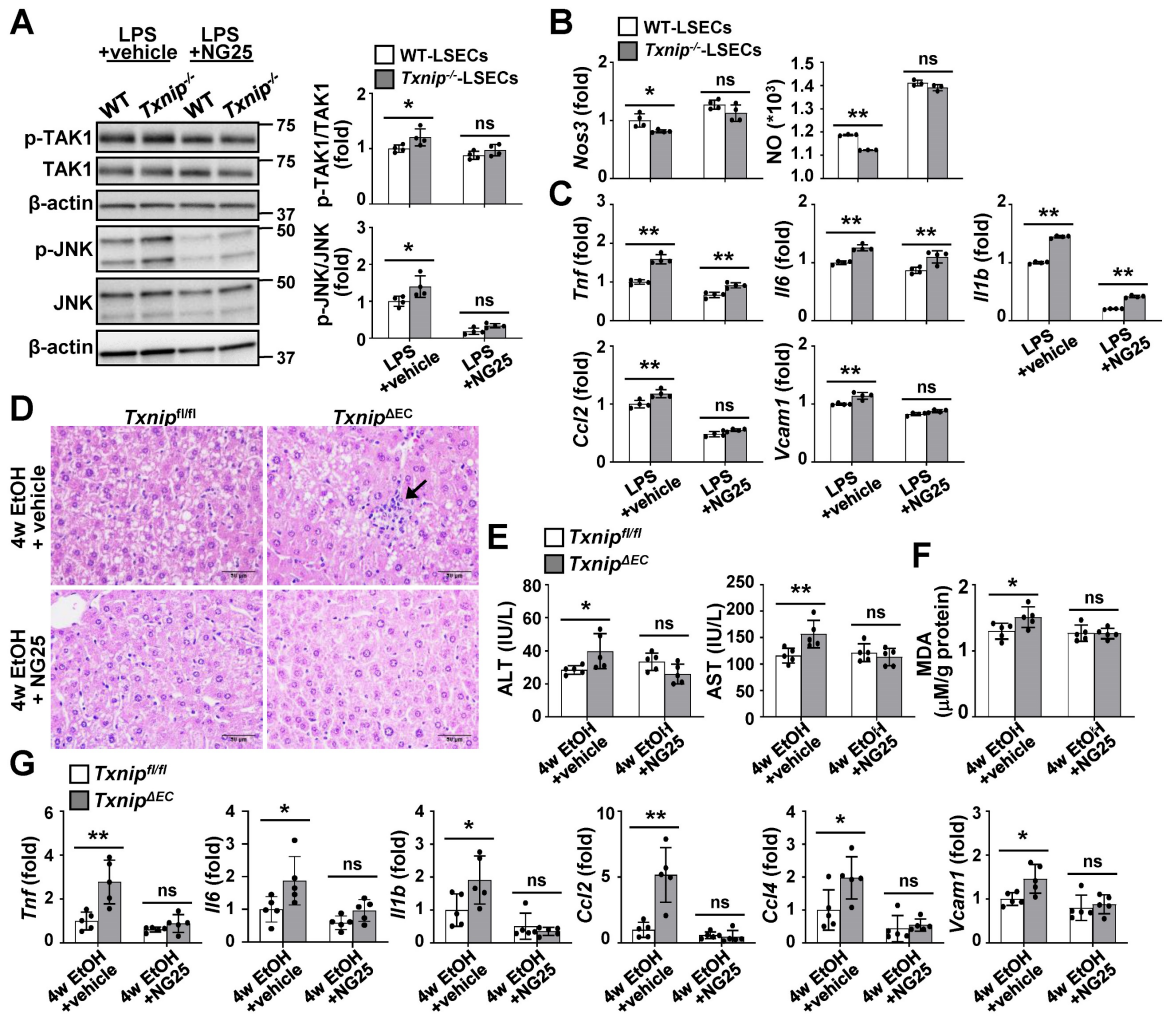
** TAK1 inhibition restores eNOS levels and ameliorates ethanol-induced liver injury, oxidative stress, and inflammation caused by *Txnip* deficiency in LSECs. (A-C)** LSECs isolated from *Txnip^-/-^* and WT mice were incubated with NG25 (500 nM) for 3 h, incubated with LPS (0.75 μg/ml) for 0.5 or 6 h, and subjected to western blot analysis (A), qRT-PCR analysis (B and C), and NO assay (B) (n = 4). **(D-G)** *Txnip^fl/fl^* and* Txnip^ΔEC^* mice were fed a 5% ethanol diet with NG25 (5 mg/kg/day) for 4 weeks (4w EtOH) and subjected to H&E staining (D), blood chemistry analysis (E), oxidative stress analysis (F), and qRT-PCR analysis (G) (n = 5). Scale bars, 50 μM. Data are presented as means ± SD. *p < 0.05; **p < 0.01. “ns” stands for “not significant.”

To further clarify whether TAK1 mediated the effects of TXNIP on ALD development, TAK1 activity was blocked in LSECs using a specific TAK1 inhibitor. Compared with the vehicle control group, NG25 treatment suppressed the LPS-induced phosphorylation of TAK1 and JNK in *Txnip*-deficient LSECs (Fig. 6A). In addition, NG25 reversed the downregulation of *Nos3* and NO production and the upregulation of proinflammatory cytokines and *Vcam1* in LPS-treated *Txnip*-deficient LSECs (Fig. 6B and C). The requirement of TAK1 for TXNIP function was further validated *in vivo*. *Txnip^fl/fl^* and *Txnip^ΔEC^* mice were fed an ethanol diet accompanied with NG25. The ethanol diet-induced increases in liver injury (liver histology and serum ALT and AST levels), oxidative stress (MDA), and cytokine and adhesion molecule levels (*Tnf*, *Il6*, *Il1b*, *Ccl2*, *Ccl4*, and *Vcam1*) in *Txnip^ΔEC^* mice were reduced to levels similar to those in *Txnip^fl/fl^* mice (Fig. 6D-G). Collectively, these results indicate that TAK1 mediates the protective effects of TXNIP during ALD development.

### LSEC capillarization is increased in patients with ALD and positively correlated with TXNIP expression

**Figure 7:**
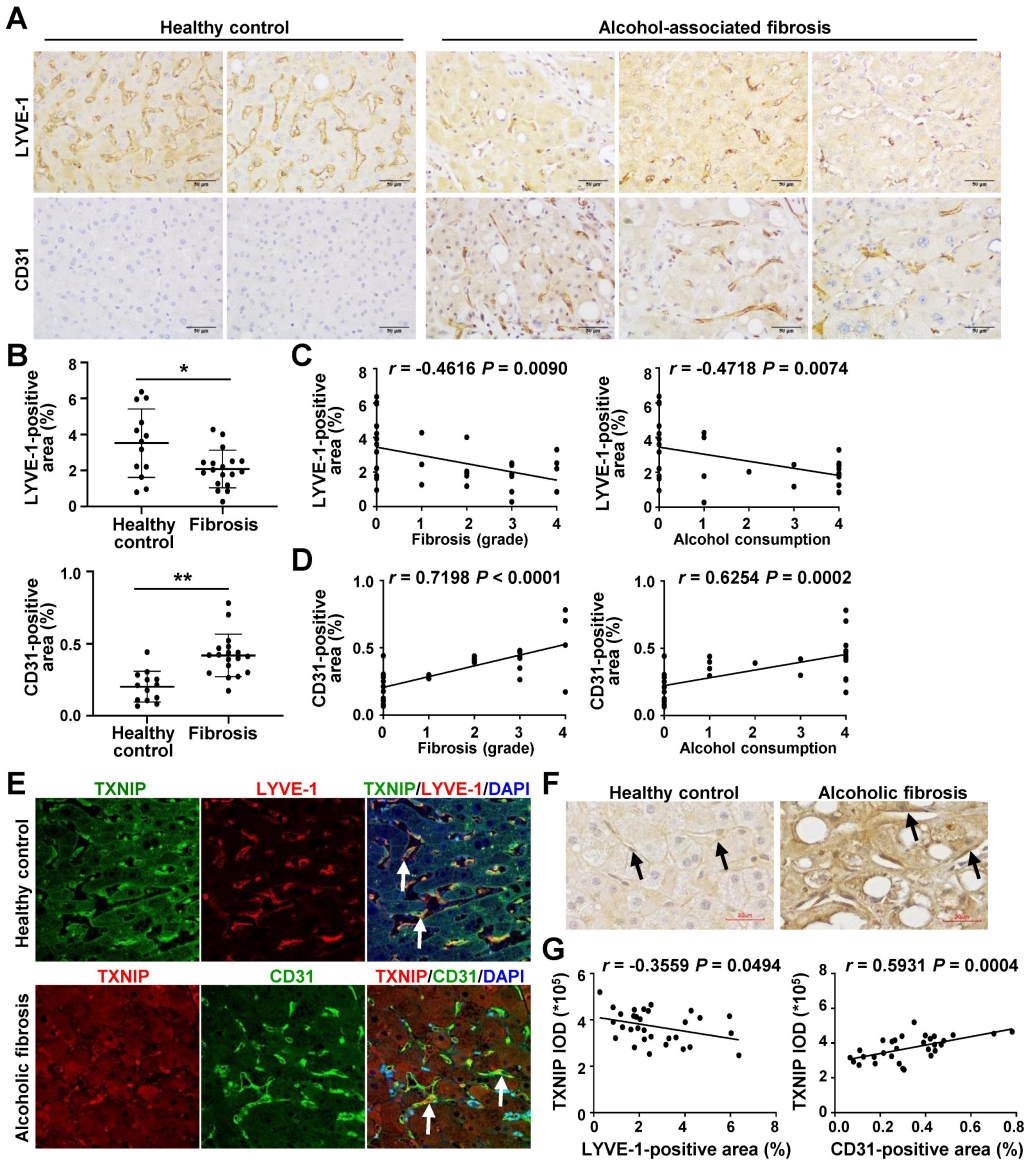
** Positive correlation between hepatic TXNIP expression and LSEC capillarization in patients with ALD. (A)** Representative image of immunohistochemical staining for LYVE-1 and CD31 in liver sections from healthy controls (n = 13) and in patients with alcohol-associated fibrosis (n = 18). Scale bars, 50 μM. **(B)** LYVE-1- and CD31-positive areas. **(C)** Negative correlation of hepatic LYVE-1 expression and fibrosis grade or alcohol consumption. **(D)** Positive association of hepatic CD31 expression and fibrosis grade or alcohol consumption. Alcohol consumption was defined as follows: Heavy (grade 4) > Moderate (grade 3) > Social (grade 2) > Occasional (grade 1) > None (grade 0).** (E)** Immunofluorescence staining of liver sections. Note the colocalization of TXNIP and LYVE-1 or CD31 (arrows). Original magnification, ×400. **(F)** Immunohistochemical images of TXNIP expression. Note the TXNIP-positive LSECs (arrows) in the human liver tissue. Scale bars, 30 μM. **(G)** Correlation of TXNIP expression and LYVE-1- or CD31-positive area in healthy controls and ALD patients. Data are presented as means ± SD. *p < 0.05; **p < 0.01. “ns” stands for “not significant.”

Finally, we examined the relevance of our experimental findings to patients with ALD. Consistent with previous reports 6, 26, the area positive for LYVE-1, an LSEC differentiation marker, was reduced in the liver of alcoholic patients with fibrosis compared with that in healthy controls, whereas the area positive for CD31, a capillary EC marker, was significantly increased (Fig. 7A and B). LYVE-1 expression was negatively correlated with fibrosis grade and ethanol intake, whereas CD31 expression was positively correlated with these parameters (Fig. 7C and D).

Similar to our in *vivo* and *in vitro* results, co-IF staining showed that TXNIP was coexpressed with LYVE-1 in healthy controls. However, patients with alcoholic fibrosis exhibited colocalization of TXNIP and CD-31 (Fig. 7E). Notably, IHC staining revealed strong immune positivity for TXNIP in LSECs of patients with alcoholic fibrosis, but weak staining in healthy controls (Fig. 7F). Examination of the correlation between TXNIP and LYVE-1 or CD31 in human ALD tissues showed that TXNIP expression was negatively and positively correlated with LYVE-1- and CD31-positive area, respectively (Fig. 7G).

## Discussion

Here, we found that TXNIP was upregulated in the livers of patients with ALD compared with that in healthy controls and mice subjected to ethanol feeding. TXNIP was overexpressed mainly in LSECs, and mice genetically lacking *Txnip* in ECs exhibited accelerated liver injury, hepatitis, fibrosis, and HCC development following ethanol feeding. The increased risk of alcohol-induced hepatocarcinogenesis in *Txnip^ΔEC^* mice is likely due to LSEC capillarization, dysfunction, and a proinflammatory phenotype. Further experiments showed that TXNIP interacted with TAK1, ultimately suppressing TAK1 activation and downstream JNK signaling. Inhibition of TAK1 activation restored eNOS levels and NO production and inhibited the release of proinflammatory mediators, further contributing to decreased liver injury and inflammation in ethanol-fed *Txnip^ΔEC^* mice.

The alcoholic liver injury is most widely modeled by *ad libitum* feeding of mice with the Lieber-DeCarli liquid diet containing 5% ethanol for 4-8 weeks 21; however, this model, without the addition of a secondary insult, only induces mild steatosis, slight elevation of serum ALT levels and little or no inflammation 27-32. Stepwise feeding of the Lieber-DeCarli ethanol diet up to 12 weeks induces remarkable fatty liver but only mild elevation of serum ALT levels 33, 34. Consistent with these results, *Txnip^fl/fl^* mice showed hepatic lipid accumulation after 4% ethanol feeding for 1 year, but mild increases in serum ALT levels and inflammatory cell infiltration. However, *Txnip^ΔEC^* mice exhibited remarkable upregulation of serum ALT and AST levels, proinflammatory cytokines, fibrosis, and HCC development, indicating that endothelial-specific deletion of *Txnip* exacerbates alcohol-induced liver diseases.

In recent years, numerous studies have reported altered LSEC phenotypes and functions in ALD. Biopsy specimens from patients with alcoholism show significantly fewer fenestrations and lower endothelial porosity than those obtained from non-alcoholics, indicating that sinusoids undergo capillarization during alcoholic liver injury 6, 26. Sinusoidal capillarization is also observed in liver biopsies of patients with alcoholism with mild fibrosis and increases as fibrosis progresses to cirrhosis 26. Here, we observed greater LSEC capillarization in patients with ALD than in healthy controls. In addition, increased expression of capillary EC markers in human ALD correlated with elevated TXNIP expression. The role of TXNIP in ECs has been previously reported. Overexpression of TXNIP reduces eNOS phosphorylation and NO generation, thus impairing endothelial function, whereas its knockdown rescues endothelial dysfunction by restoring eNOS expression 19, 20. TXNIP promotes inflammation by increasing monocyte adhesion in ECs 16, whereas silencing TXNIP prevents LPS-induced inflammatory response, apoptosis, and oxidative damage 35. In contrast, we found that *Txnip* deletion in LSECs induced capillarization, reduced eNOS levels and NO production, and increased the release of proinflammatory mediators and adhesion molecules, accompanied by accelerated alcohol-induced hepatocarcinogenesis. This discrepancy may be attributed to the unique phenotype of LSECs, which differ morphologically and functionally from capillary ECs in the presence of typical fenestrations, clustering in sieve plates, and the absence of a basement membrane 3. Given that overexpression of TXNIP inhibits sinusoidal capillarization and stimulates eNOS level and NO production, the increased TXNIP expression in LSECs of patients with ALD may be a compensatory mechanism to protect liver from alcohol-induced damage. This is consistent with the findings of our previous study, which showed that TXNIP expression is increased in hepatocytes of MASLD patients and elevated TXNIP serves a protective function to ameliorate steatohepatitis by promoting autophagy and fatty acid-oxidation 13.

Our systematic RNA-seq analyses showed that the MAPK signaling cascade was closely associated with *Txnip* loss in LSECs. *Txnip* deficiency in LSECs increased JNK activation, which was accompanied by decreased eNOS levels, NO production, and an enhanced inflammatory response. Consistently, higher JNK phosphorylation was observed in ethanol-fed *Txnip^ΔEC^* mice than in *Txnip^fl/fl^* mice. Activation of p38 MAPK and ERK did not change in ethanol-fed *Txnip^ΔEC^* mice, indicating that TXNIP protects against hepatic ALD by regulating JNK signaling. Previous studies have demonstrated that JNK activation contributes to endothelial dysfunction by inhibiting eNOS phosphorylation 36, 37, whereas JNK inhibition restores eNOS synthase activation and endothelial function 38. JNK activation also upregulates the expression of inflammatory mediators and adhesion molecules, promoting the development of inflammatory liver diseases 39-41. In addition, preclinical studies on the use of JNK-specific inhibitors found them to exert protective effects by attenuating endothelial dysfunction and inflammation 42, 43. We further explored the direct interaction between the MAPK pathway and TXNIP and found that TXNIP interacted with TAK1 and regulated its activation in LSECs. A previous study has shown that TXNIP interacts with the 35-291 region of TAK1 in HEK 293 cells and inhibits TAK1 activity by interfering with the formation of the TAK1 and TAK1-binding protein 1 (TAB1) complex 44. Although the mechanism by which TXNIP regulates TAK1 in LSECs requires further investigation, our results show that TXNIP interacts with TAK1, thereby, attenuating TAK1 phosphorylation and subsequent JNK signaling in LSECs. The potential role of TAK1 in various liver diseases, including ischemia-reperfusion injury, MASLD, metabolic dysfunction-associated steatohepatitis (MASH), and HCC, is well defined 45-48. The suppression of TAK1 and its downstream signaling significantly promotes liver injury, inflammation, fibrosis, and tumor formation 45, 48. While TAK1 hyperactivation induces hepatic steatosis, inflammation, and fibrosis, TAK1 activity suppression alleviates the progression of these diseases 45, 47, 49. In the present study, TAK1 inhibition attenuated JNK activation, restored eNOS levels and NO production, and downregulated cytokines and adhesion molecules in *Txnip*-deficient LSECs. Subsequently, ethanol-induced liver injury and inflammation were ameliorated in *Txnip^ΔEC^* mice following treatment with a TAK1 inhibitor. These results demonstrate that TXNIP plays a protective role in ALD by suppressing TAK1 activation and downstream JNK signaling.

The conservation of the LSEC phenotype and prevention of capillarization may reduce the risk of progression from inflammation and fibrosis to irreversible cirrhosis and HCC 50. Approaches that maintain LSEC differentiation and function include restoration of their fenestrated phenotype via bone morphogenic protein 9 (BMP9) and GATA4 51, 52, reconstitution of VEGF/NO/sGC signaling 53, 54, and reestablishment of normal hedgehog signaling 55. The present study shows that TXNIP maintains the LSEC phenotype and function through interactions with TAK1 and JNK signaling, which protects against ALD progression. Our findings provide insights into LSEC-mediated ALD development and suggest that enhancing TXNIP expression may constitute a potential strategy to confer protection against ALD.

## Supplementary Material

Supplementary figures and tables.

## Abbreviations

ALD: alcohol-associated liver disease
ALT: alanine aminotransferase
AST: aspartate aminotransferase
CCL4: C-C motif chemokine 4
COL1α1: collagen 1 α1
DEGs: differentially expressed genes
ECs: endothelial cells
eNOS: endothelial nitric oxide synthase
ERK: extracellular signal-regulated kinase
Edn1: endothelin-1
HCC: hepatocellular carcinoma
HSCs: hepatic stellate cells
ICAM-1: intercellular adhesion molecule 1
IL: interleukin
JNK: c-Jun N-terminal kinase
KCs: Kupffer cells
KEGG: Kyoto encyclopedia of genes and genomes
LPS: lipopolysaccharide
LSECs: liver sinusoidal endothelial cells
LYVE-1: lymphatic vessel endothelial hyaluronan receptor 1
CCL: C-C motif chemokine ligand
MASLD: metabolic dysfunction-associated steatotic liver disease
NO: nitric oxide
TAK1: transforming growth factor β-activated kinase 1
TGF-β: transforming growth factor-β
TIMP-1: tissue inhibitor of metalloproteinases-1
TNF-α: tumor necrosis factor-α
TXNIP: thioredoxin-interacting protein
α-SMA: alpha-smooth muscle actin
VAP-1: vascular adhesion protein 1
VCAM-1: vascular cell adhesion protein 1
WT: wild-type
