# Supplementary material for: TXNIP in liver sinusoidal endothelial cells ameliorates alcohol-associated liver disease via nitric oxide production
Source: Int J Biol Sci. 2024 Jan 1;20(2):606–20. doi: 10.7150/ijbs.90781 (PMC10758096; doi:10.7150/ijbs.90781)
Supplement: Supplementary file 1 — Supplementary figures and tables. [file ijbsv20p0606s1.pdf]

TXNIP in liver sinusoidal endothelial cells ameliorates alcohol-associated liver disease via nitric oxide production

Eunhye Jung[1,*], Eun Bok Baek[1,*], Eun-Ju Hong[1], Jee Hyun Kang[1], Suyoung Park[1], Sehee Park[2], Eui-Ju Hong[1], Young-Eun Cho[3], Je-Won Ko[1], Young-Suk Won[2,#], and Hyo-Jung Kwon[1,#]

[1]*College of Veterinary Medicine, Chungnam National University, Daejeon 34134, Republic of Korea*

[2]*Laboratory Animal Resource Center, Korea Research Institute of Bioscience and Biotechnology, Chungbuk 28116, Republic of Korea*

[3]*Andong National University, Andong 36729, Republic of Korea*

Table of Contents:

Supplementary Figures……………………………………………………………….2

Supplementary Tables………………………………………………………………..9

Supplementary References…………………………………………………….......13

Supplementary Figures

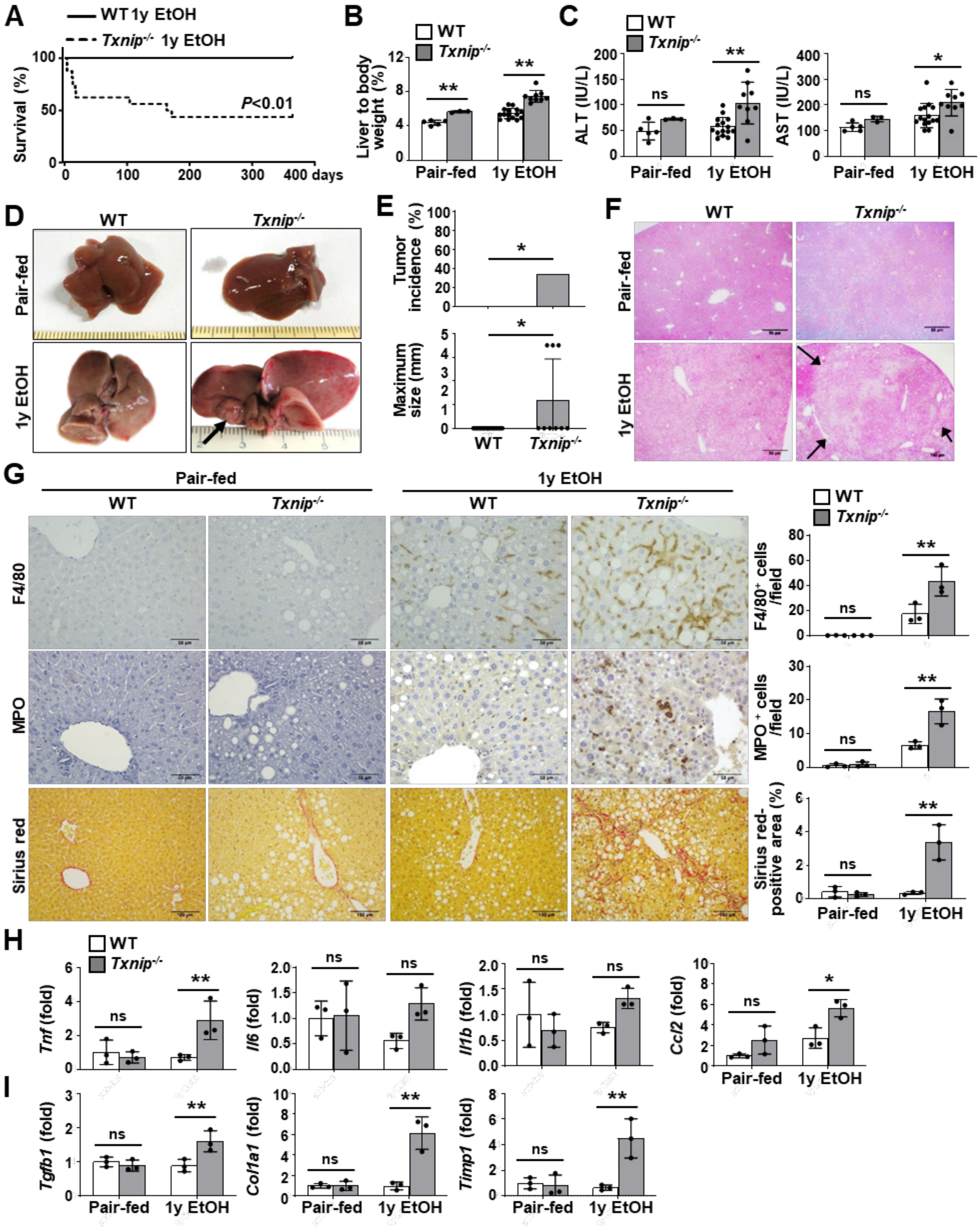

Fig. S1. A lack of *Txnip* increases ethanol-induced liver injury, inflammation, fibrosis, and HCC development. *Txnip*$^{-/-}$ and WT mice were fed a control or 4% ethanol diet for 1 year (1y EtOH). **(A)** Survival rate after 1y EtOH of ethanol treatment (WT, n = 16; *Txnip*$^{-/-}$, n = 16). **(B)** Liver-to-body weight (pair-fed WT, n = 5; pair-fed *Txnip*$^{-/-}$, n = 3; 1y EtOH WT, n = 15; 1y EtOH *Txnip*$^{-/-}$, n = 9). **(C)** Serum levels of ALT and AST (pair-fed WT, n = 5; pair-fed *Txnip*$^{-/-}$,

n = 3; 1y EtOH WT, n = 15; 1y EtOH *Txnip*$^{-/-}$, n = 9). **(D)** Representative gross findings of livers. Arrow indicates liver tumor. **(E)** The incidence and maximum size of tumor after 1y EtOH feeding (WT, n = 15; *Txnip*$^{-/-}$, n = 9). **(F)** H&E staining of livers. Note the tumor (arrows) compressing adjacent normal liver tissue in *Txnip*$^{-/-}$ mice. Scale bars, 50 μM. **(G)** Representative immunohistochemistry of F4/80 and MPO and Sirius red staining (n = 3). Scale bars, 100 μM or 50 μM. **(H)** Relative mRNA levels of inflammatory response genes (n = 3). **(I)** Relative mRNA levels of genes related to fibrosis (n = 3). Values represent means ± SD. *p < 0.05; **p < 0.01. “ns” stands for “not significant”.

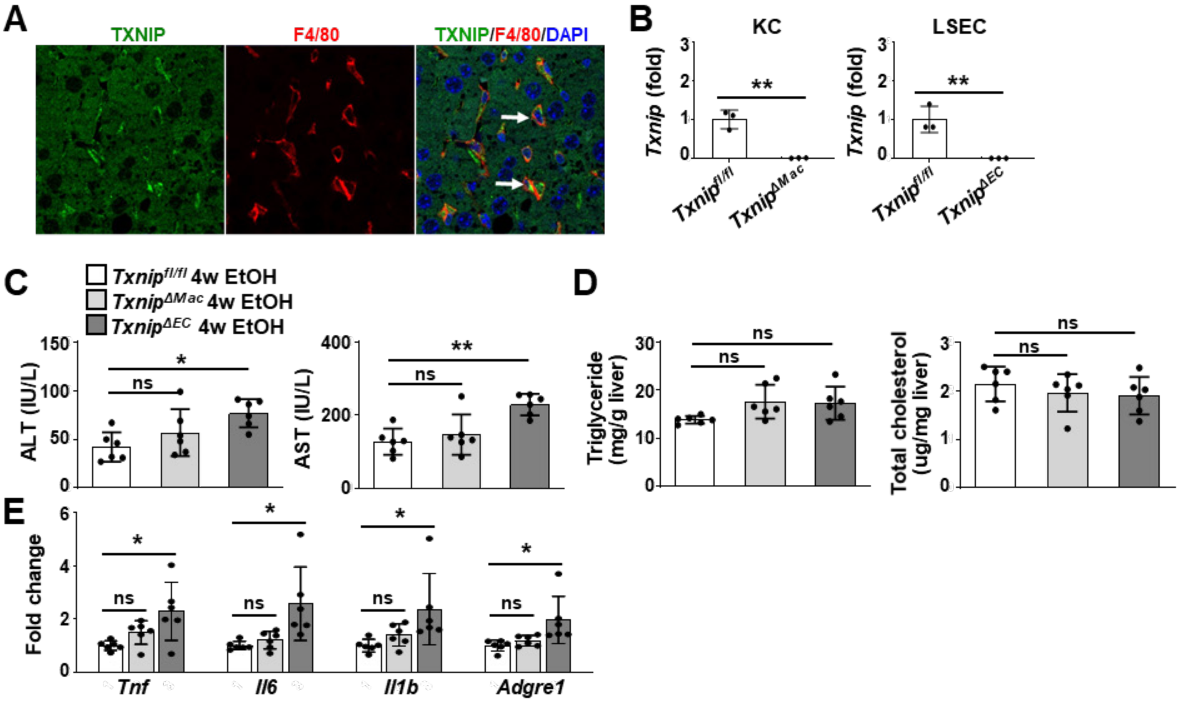

Fig. S2. LSEC-specific *Txnip* knockout increases ethanol-induced liver diseases. (A) Immunofluorescence staining of WT mice. Note the co-localization of TXNIP and F4/80 (arrows). Original magnification, ×400. **(B)** Validation of KCs and LSECs *Txnip* deletion in $Txnip^{\Delta Mac}$ and $Txnip^{\Delta EC}$ mice. KCs and LSECs were isolated from $Txnip^{fl/fl}$, $Txnip^{\Delta Mac}$, and $Txnip^{\Delta EC}$ mice (n = 3). **(C–E)** $Txnip^{fl/fl}$, $Txnip^{\Delta Mac}$, and $Txnip^{\Delta EC}$ mice were fed a 5% ethanol diet for 4 weeks (4w EtOH) and analyzed for serum ALT and AST (C), hepatic triglyceride and cholesterol (D), and relative hepatic mRNA levels of inflammatory response genes (E) (n = 6). Data are shown as means ± SD. *$p < 0.05$; **$p < 0.01$. "ns" stands for "not significant".

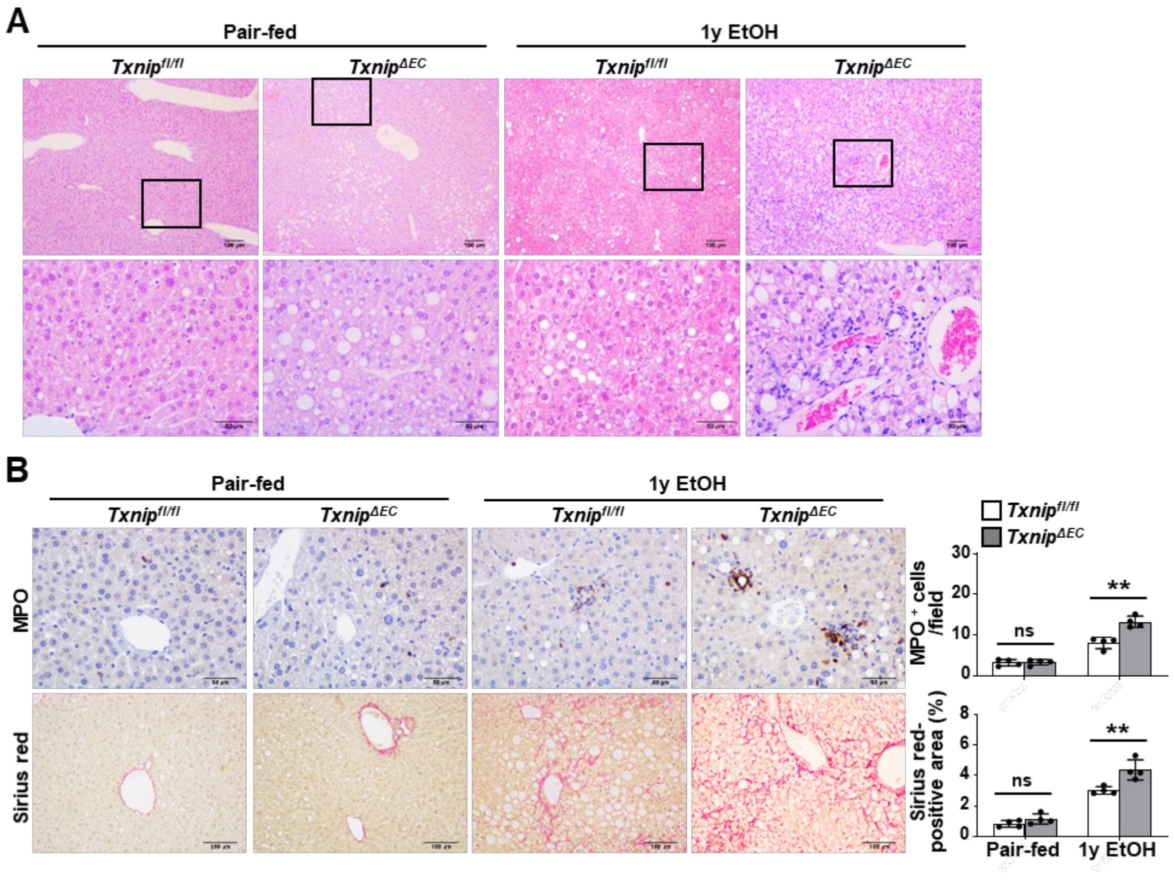

Fig. S3. LSEC-specific *Txnip* knockout increases ethanol-induced liver injury, inflammation, and fibrosis. *Txnip*$^{fl/fl}$ and *Txnip*$^{\Delta EC}$ mice fed a control or 4% ethanol diet for 1 year (1y EtOH). **(A)** H&E staining. **(B)** Representative immunohistochemistry of MPO and Sirius red staining (n = 4). Scale bars, 100 µM or 50 µM. Values represent means ± SD. *p < 0.05; **p < 0.01. "ns" stands for "not significant".

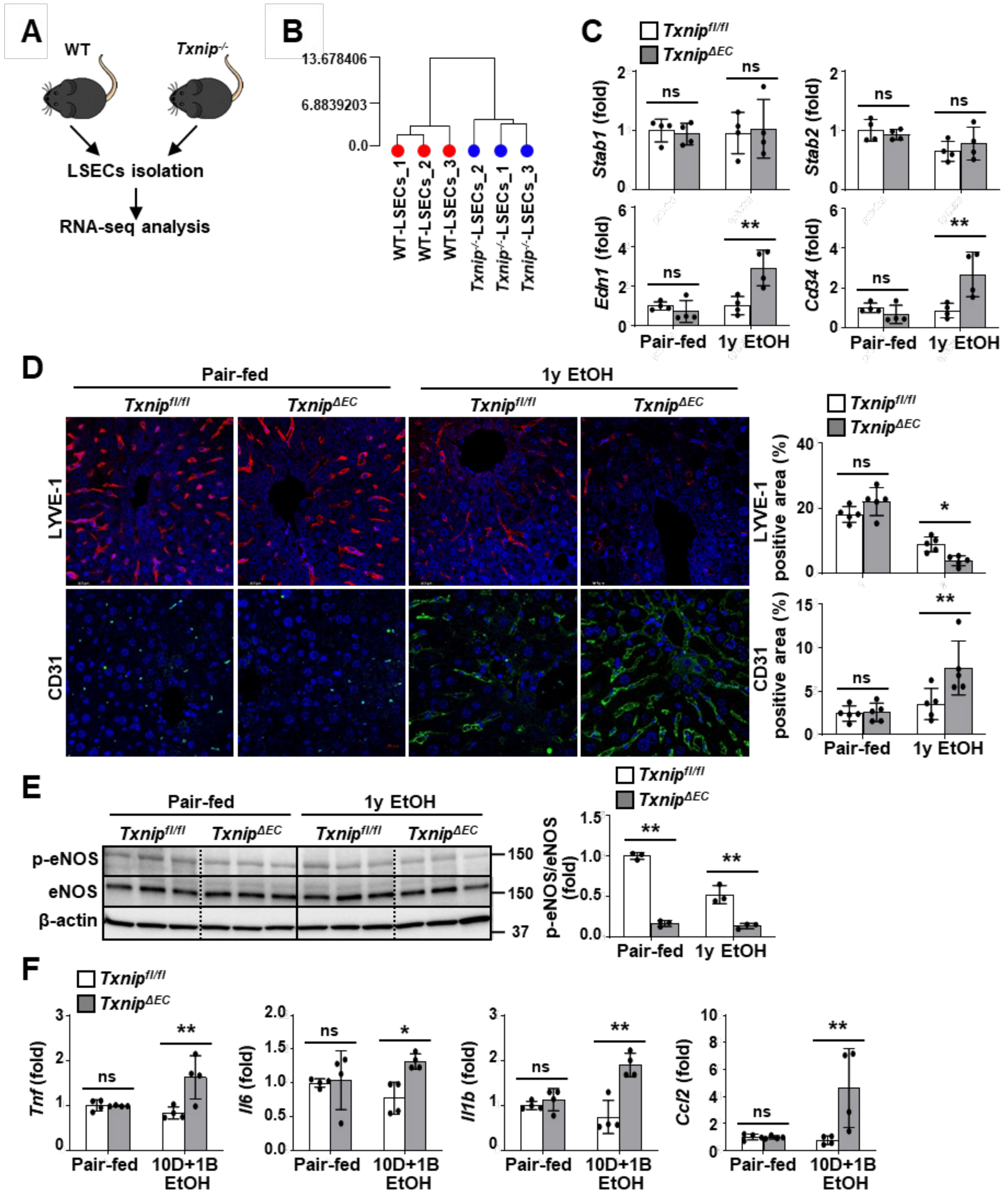

Fig. S4. Endothelial TXNIP regulates LSEC capillarization and dysfunction. (A) RNA-seq construction plan for LSECs isolated from WT and *Txnip*$^{-/-}$ mice (n = 3). **(B)** Hierarchical clustering dendrogram showing the distribution profiles of RNA-seq results. **(C)** mRNA levels of hepatic genes involved in LSEC differentiation (n = 4). *Txnip*$^{fl/fl}$ and *Txnip*$^{\Delta EC}$ mice were fed a liquid control diet or 4% ethanol diet for 1 year (1y EtOH). **(D)** Immunofluorescence staining of LYVE-1 and CD31 (n = 5). *Txnip*$^{fl/fl}$ and *Txnip*$^{\Delta EC}$ mice were fed a control or 4% ethanol diet for 1 year (1y EtOH). LYVE-1- or CD31-positive areas are shown on the right. Original magnification, ×400. **(E)** The expression levels of phospho- and total-eNOS (n = 3). *Txnip*$^{fl/fl}$ and *Txnip*$^{\Delta EC}$ mice were fed either a liquid control diet or 4% ethanol diet for 1 year (1y EtOH).

(F) The level of cytokines. LSECs were isolated from *Txnip*$^{fl/fl}$ and *Txnip*$^{\Delta EC}$ mice after 10 day plus gavage (10D+1B EtOH) and subjected to qRT-PCR (n = 4). Data are shown as means ± SD. $^{*}p < 0.05$; $^{**}p < 0.01$. “ns” stands for “not significant”.

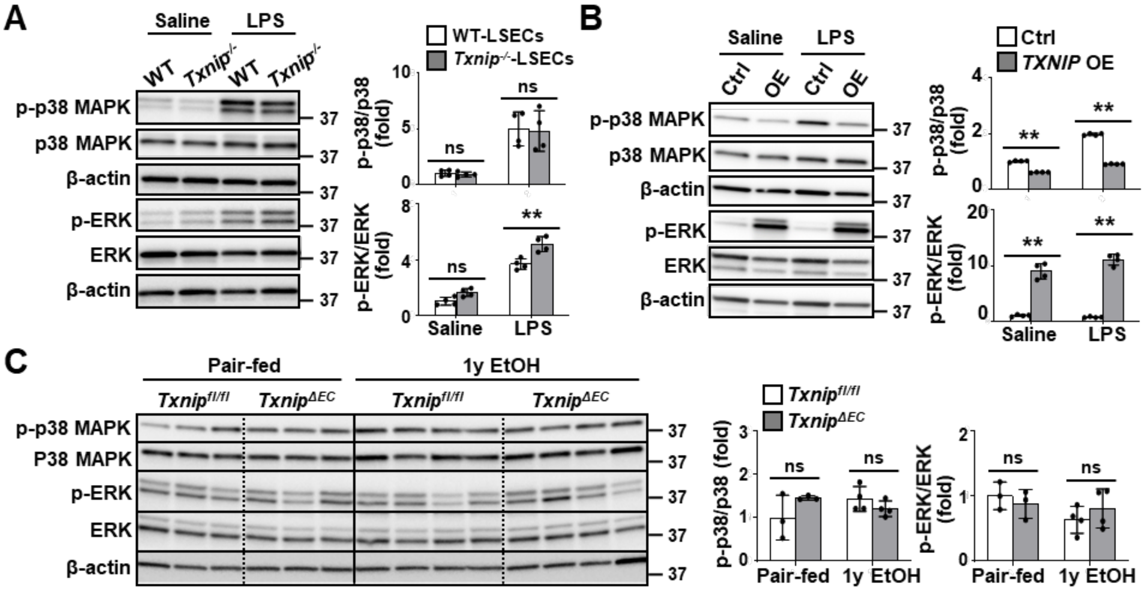

Fig. S5. The effects of TXNIP on p38 MAPK and ERK in LSECs. (A) Protein expression of p38 MAPK and ERK (n = 4 biological replicates). LSECs isolated from WT and *Txnip*$^{-/-}$ mice were incubated with saline or LPS (0.75 µg/ml) for 30 min. **(B)** Western blot analysis (n = 4 biological replicates). Control (Ctrl) and *TXNIP*-overexpressing (*TXNIP* OE) TMNK-1 cells were incubated with saline or LPS (0.75 µg/ml) for 30 min. **(C)** Western blot analysis of liver tissues (pair-fed, n = 3; EtOH, n = 4). *Txnip*$^{fl/fl}$ and *Txnip*$^{\Delta EC}$ mice were fed a liquid control diet or 4% ethanol diet for 1 year (1y EtOH). Data are shown as means ± SD. *$p < 0.05$; **$p < 0.01$. “ns” stands for “not significant”.

Supplementary Tables
Table S1. Donor information

Donor information with normal liver histology (n = 13)

Pathology	Gender	Age (yr)	Ethnicity	Macro Fat (%)	BMI (kg/m^2)	Alcohol Consumption
Normal, Down syndrome	Male	41	African American	0	24.8	None
Normal	Male	26	Caucasian	0	24.5	None
Normal	Female	24	African American	0	54.6	None
Normal	Female	58	African American	0	31.3	None
Normal	Male	18	Caucasian	0	25.3	None
Normal	Male	21	Caucasian	1	24.6	None
Normal, coronary artery disease	Male	50	Asian	<1	26.4	None
Normal	Male	43	Caucasian	0	30.0	None
Normal	Male	63	Caucasian	0	27.4	None
Normal	Female	49	Caucasian	<1	30.3	None
Normal, cerebral palsy	Female	56	Asian	0	37.9	None
Normal	Male	56	Caucasian	0	13.7	None
Normal	Male	41	Caucasian	0	25.7	None

Donor information with liver steatosis with a history of alcohol use (n = 19)

Pathology	Gender	Age	Ethnicity	Macro Fat (%)	BMI	Alcohol Consumption
Steatosis with history of alcohol	Male	32	Caucasian	10-15	32.9	Heavy
Steatosis with history of alcohol	Male	84	Caucasian	50	28.8	Occasional
Steatosis with history of alcohol	Male	38	Caucasian	60	37.2	Social
Steatosis with history of alcohol	Male	21	Caucasian	90	23.7	Moderate
Steatosis with history of alcohol	Male	44	Hispanic	60	23.1	Occasional
Steatosis with history of alcohol	Male	40	Caucasian	50	22.1	Heavy
Steatosis with history of alcohol	Male	40	Native American	40	21.5	Moderate
Steatosis with history of alcohol	Male	55	African American	10	34.4	Social
Steatosis with history of alcohol	Male	49	Caucasian	70	24.4	Moderate
Steatosis with history of alcohol	Female	47	Caucasian	90	27.4	Heavy
Steatosis with history of alcohol	Female	55	Caucasian	60	48.1	Heavy
Steatosis with history of alcohol	Male	47	Caucasian	25	40.3	Social
Steatosis with history of alcohol	Female	63	Caucasian	80	36.7	Occasional
Steatosis with history of alcohol	Female	60	Caucasian	50	45.4	Heavy
Steatosis with history of alcohol	Male	49	Caucasian	10	43.3	Occasional
Steatosis with history of alcohol	Female	46	Caucasian	10	48.4	Occasional
Steatosis with history of alcohol	Male	53	Hispanic	5-10	33.5	Moderate
Steatosis with history of alcohol	Male	38	Caucasian	30-40	31.9	Occasional
Steatosis with history of alcohol	Male	58	Caucasian	20	32.1	Occasional

Donor information with liver fibrosis with a history of alcohol use (n = 18)

Pathology/fibrosis*	Gender	Age (yr)	Ethnicity	Macro Fat (%)	BMI (kg/m^2)	Alcohol Consumption
Cirrhosis (4)	Female	56	Caucasian	5	23.1	Heavy
Cirrhosis (4)	Female	52	Caucasian	20	24.2	Heavy
Cirrhosis (4)	Female	55	Caucasian	5	20.4	Heavy
Cirrhosis (4)	Male	55	Caucasian	40	25.1	Heavy
Steatohepatitis, ballooned hepatocytes, bridging fibrosis (3)	Female	59	Caucasian	80	23.4	Heavy
Steatohepatitis, perisinusoidal fibrosis, bridging fibrosis (3)	Female	36	Caucasian	50	17.6	Heavy
Steatohepatitis, ballooned hepatocytes, bridging fibrosis (3)	Female	64	Caucasian	50	17.2	Heavy
Steatohepatitis, bridging fibrosis (3)	Female	42	Caucasian	10	25.2	Heavy
Steatohepatitis, bridging fibrosis (3)	Female	49	Caucasian	3	30.2	Occasional
Steatosis, moderate, perisinusoidal, portal/periportal (2)	Male	84	Caucasian	50	28.8	Occasional
Steatosis, mild, perisinusoidal, portal/periportal (1)	Male	56	Caucasian	10	48.2	Heavy

NASH, occasional ballooned hepatocytes, focal perisinusoidal, portal/periportal (2)	Male	90	Caucasian	90	40.7	Occasional l
Steatohepatitis, ballooned hepatocytes, bridging fibrosis (3)	Male	40	Caucasian	40	32.2	Heavy
Steatosis, mild, focal perisinusoidal, portal/periportal (2)	Female	25	Caucasian	25	42.0	Moderate
NASH, scattered ballooned hepatocytes, portal/periportal (1)	Female	30	Caucasian	30	30.2	Occasional
Steatosis, mild, diffused portal/periportal (1)	Male	29	Caucasian	30	33.8	Moderate
Steatosis, moderate, focal perisinusoidal, portal/periportal (2)	Male	38	Caucasian	50	37.2	Social
Steatosis, severe, very mild portal/periportal (1)	Male	80	Caucasian	80	37.3	Heavy

Alcohol consumption terms (listed highest to lowest):
Heavy (grade 4) > Moderate (grade 3) > Social (grade 2) > Occasional (grade 1) > None (grade 0)
*Fibrosis stage was assigned according to a previous study [1].

Table S2. Antibodies used for IHC, IF, and western blot

Name	Citation	Supplier	Cat. No.
anti-β-actin	PMID: 36187165	Sigma	A5441
anti-CD31	PMID: 32122268	Abcam	ab28364
	PMID: 33664870	Abcam	ab56299
anti-p-eNOS	PMID: 11714864	Cell Signaling Technology	9570
anti-p-ERK1/2	PMID: 15187187	Cell Signaling Technology	4370
anti-F4/80	PMID: 32157317	Abcam	ab6640
anti-p-JNK	PMID: 10207617	Cell Signaling Technology	4668
anti-LYVE1	PMID: 7691670	R&D Systems	AF2089
	PMID: 7691670	R&D systems	BAF2125
anti-MPO	PMID: 34454169	Abcam	ab9535
anti-p-p38 MAPK	PMID: 7923353	Cell Signaling Technology	4511
anti-p-TAK1	PMID: 8533096	Cell Signaling Technology	9339
anti-total eNOS	PMID: 11714864	Cell Signaling Technology	32027
anti-total ERK1/2	PMID: 15187187	Cell Signaling Technology	4696
anti-total JNK	PMID: 10207617	Cell Signaling Technology	9252
anti-total p38 MAPK	PMID: 7923353	Cell Signaling Technology	9212
anti-total TAK1	PMID: 8533096	Cell Signaling Technology	5206
anti-TXNIP	PMID: 32583421	Abcam	ab188865
Alexa Fluor™ Plus 488-goat anti-rabbit	PMID: 32142286	Invitrogen	A32731
Alexa Fluor™ Plus 555 donkey anti-goat	PMID: 33633238	Invitrogen	A32816
Alexa Fluor™ Plus 555 goat anti-mouse	PMID: 32688332	Invitrogen	A21424
goat anti-rabbit IgG	PMID: 32879487	Vector Laboratories	PK-6101
rabbit anti-rat IgG	PMID: 36050303	Vector Laboratories	PK-6104

Table S3. Primers used for qRT-PCR-based gene expression analysis

Name	Forward (5' – 3')	Reverse (5' – 3')
Human		
CD34	AGC TGT GCG GAG TTT AAG AAG GAC	TCA CCT CAG ACT GGG CAA GGA G
EDN1	GGT CTA ATG TGT CAG CAG TA	CCT ATA CCA TTG TGT CTC CAA
STAB1	AAC GCC ACC CTC CTA AGT G	GCT AAC CAC AAC TGT CCC TG
GAPDH	AAA TCC CAT CAC CAT CTT CCA	AAA TGA GCC CCA GCC TTC TC
STAB2	AGA TCA GCA ATG GAG GTT GC	GAT TTC CAG GCA CAC AAT GC
Mouse		
Ccl4	CCT GAC CAA AAG AGG CAG AC	AAG AAG AGG GGC AGG AAA TC
Col1a1	GCT CCT CTT AGG GGC CAC T	ATT GGG GAC CCT TAG GCC AT
Col3a1	CTG AAG ATG TCG TTG ATG TG	CTG ATC CAT ATA GGC AAT ACT G
Col4a1	TCC GGG AGA GAT TGG TTT CC	CTG GCC TAT AAG CCC TGG T
Cd34	CTT CTG CTC CGA GTG CCA TT	AAC TCC TCA CAA CTA GAT GCT TCA
Nos3	CAA CGC TAC CAC GAG GAC TT	CTC CTG CAA AGA AAA GCT CTG
Edn1	ACC GTA TGG ACT GGG AGG TT	GGT GAG CGC ACT GAC ATC TA
Adgre1	AAC ATG CAA CCT GCC ACA AC	TTC ACA GGA TTC GTC CAG GC
Gapdh	TGC ACC ACC AAC TGC TTA G	GGA TGC AGG GAT GAT GTT C
Icam1	CCA TCA CCG TGT ATT CGT TT	GAG GTC CTT GCC TAC TTG CT
Il6	TCC ATC CAG TTG CCT TCT TG	TTC CAC GAT TTC CCA GAG AAC
Il1b	AAA AAA GCC TCG TGC TGT CG	GTC GTT GCT TGG TTC TCC TTG
Laminin	GAA AGG AAG ACC CGA AGA AAA	CCA TAG GGC TAG GAC ACC AAA
Ccl2	GCA TCC ACGTGTTGGTCA	CTC CAG CCT ACT CAT TGG GAT C
Stab1	TCA CTG TCC CCA CAC TAC TTT	TGT CGC AAC GTT TAG ACC GTA
Stab2	AACTGGCAGTGATGTCGGAG	GGA TTG GAG AAA CAG CTC CC
Tgfb1	TTG CTT CAG CTC CAC AGA GA	TGG TTG TAG AGG GCA AGG AC
Timp1	GCA ACT CGG ACC TGG TCA TAA	CGG CCC GTG ATG AGA AAC T
Tnf	AAG CCT GTA GCC CAC GTC GTA	AGG TAC AAC CCA TCG GCT GG
Vap1	GTG GTC AGA TCC GTG TCT ACC TT	CCT GTG GCG TGG AAT TTG A
Vcam1	TCT TAC CTG TGC GCT GTG AC	TTA CTG GAT CTT CAG GGA ATG AG

Supplementary Reference

1. Brunt EM, Janney CG, Di Bisceglie AM, Neuschwander-Tetri BA, Bacon BR. Nonalcoholic steatohepatitis: a proposal for grading and staging the histological lesions. Am J Gastroenterol. 1999; 94: 2467-74.
